# Adjunctive treatment with aripiprazole for olanzapine-induced hyperprolactinemia

**DOI:** 10.1192/j.eurpsy.2022.2003

**Published:** 2022-09-01

**Authors:** S. Bise, G. Sulejmanpasic, A. Hrnjica, Š. Šarkić-Bedak

**Affiliations:** 1 Psychiatric hospital, Women Department, Sarajevo, Bosnia and Herzegovina; 2 Clinical Centre University of Sarajevo, Psychiatric Clinic, Sarajevo, Bosnia and Herzegovina

**Keywords:** Olanzapine, Hyperprolactinemia, Amenorrhea, Aripiprazole

## Abstract

**Introduction:**

Hyperprolactinemia is a common unwanted antipsychotic-induced adverse effect, particularly in female patients, and can induce poor adherence to treatment. Aripiprazole is an antipsychotic with partial agonist activity over the dopamine D2 receptors which can be effective in reducing hyperprolactinemia in patients treated with antipsychotics.

**Objectives:**

We investigate the efficacy of adjunctive treatment with aripiprazole for olanzapine-induced hyperprolactinemia and related hormonal side effects (amenorrhea, oligomenorrhea) in female patients with schizophrenia.

**Methods:**

Eight female patients (22 to 40 years old) participated in this study with a diagnosis of schizophrenia and hyperprolactinemia-related hormonal side effects (amenorrhea, oligomenorrhea). Patients were treated with aripiprazole 10 mg/day added to a fixed olanzapine dose of 20 mg/day. Serum prolactin levels were measured at baseline and after 2, 4, 6, and 8 weeks. Symptoms and side effects were assessed using the Brief Psychiatric Rating Scale, Clinical Global Impressions Severity scale, Barnes Akathisia Scale.

**Results:**

Adjunctive treatment with aripiprazole resulted in significantly lower prolactin levels beginning at week 2. 87.5 % of patients at week 8 had prolactin levels normalize. Among 8 patients with menstrual disturbances, 75% of patients regained menstruation during the study. No significant changes were observed regarding psychopathology and adverse effect ratings.

**Conclusions:**

Adjunctive aripiprazole treatment is effective for resolving olanzapine-induced hyperprolactinemia and reinstatement of menstruation in female patients, provides significant improvement and it appears to be safe with a lower risk of metabolic syndrome, without increased risk of adverse effects.

**Disclosure:**

No significant relationships.

